# Editorial: Transitions between Consciousness and Unconsciousness

**DOI:** 10.3389/fpsyg.2018.00020

**Published:** 2018-01-24

**Authors:** Marcus Rothkirch, Morten Overgaard, Guido Hesselmann

**Affiliations:** ^1^Visual Perception Laboratory, Department of Psychiatry and Psychotherapy, Charité—Universitätsmedizin Berlin, Corporate Member of Freie Universität Berlin, Humboldt-Universität zu Berlin, and Berlin Institute of Health, Berlin, Germany; ^2^Center for Functionally Integrative Neuroscience, Aarhus University, Aarhus, Denmark

**Keywords:** consciousness, neural correlates of consciousness, unconscious processing, awareness, diversity

Over the last years, a large body of experimental data have been generated in the attempt to understand consciousness and its neural underpinnings. In this respect, particular interest has been paid to the attempt to distinguish between conscious experience and unconscious states which however may still be considered as mental states (e.g., in virtue of their representational nature). This is of course not without reason. A deep understanding of that which specifically characterizes conscious states, including neural correlates and cognitive functions, may crucially inform the ambition of understanding the relation between experience and the physical world. Nevertheless, the question has historically been challenged by the fact that consciousness is available in the first person only—not to other people, including scientists. Different methodological traditions and choices have led to quite different understandings of how conscious and unconscious states relate (e.g., Rothkirch and Hesselmann, this research topic), and diverse empirical work has been inspired and guided by various cognitive and neurobiological theories of consciousness. The very diverse viewpoints include such different positions as the idea that unconscious states are associated with the very same functional characteristics as conscious states (e.g., Hassin, 2013), and the idea that no informational state that is available for action can be completely unconscious (Overgaard and Mogensen, 2014, 2015).

The Research Topic “Transitions between consciousness and unconsciousness” is therefore devoted to this particular question, how to understand the relation and transition between consciousness and unconsciousness. It comprises 18 articles from different backgrounds, including original studies, as well as reviews and commentaries, which speaks to the multifaceted research in this field. In the following, we will provide a brief summary for each contribution.

One of the most appealing questions in the field of consciousness research is whether stimuli that cannot be consciously perceived by the observer can nevertheless influence the observer's behavior, and if, to what extent they do so. Four submissions to the research topic approached this fascinating question while focusing on different processes and behavioral outcomes. One fruitful approach to study the aforementioned unconscious influences is masked priming. Priming refers to the observation that the response to a target stimulus can be influenced by the presentation of an irrelevant prime stimulus prior to the target. In masked priming, in particular, the prime stimulus is not consciously perceived. On the basis of this paradigm, Goller et al. investigated affective priming, indicating that an incongruence between prime and target entails a negative evaluation of a neutral symbol following the target. The authors observed that such affective priming effects were stronger for unconscious than for conscious primes, which they interpreted as a misattribution of the prime-target incongruence to the unrelated neutral symbol. In a similar vein, Khalid and Ansorge used masked priming to study the subliminal processing of faces displaying disgust. Using low- and high-pass filtered faces as prime stimuli, the authors intended to identify a potential subcortical origin of the priming effect. Surprisingly, however, they found a reversed priming effect, such that a prime-target congruence led to slower reaction times. This effect was further restricted to conditions in which attention was diverted away from the prime. This points toward a unique unconscious effect of disgusting faces, which does not seem to rely on subcortical pathways. While also studying the effect of subliminal facial expressions, Winkielman and Gogolushko focused on another, “higher-level” behavioral outcome, namely the consumption of a beverage. When primed with a positive facial expression participants tended to consume more than after being primed with a negative expression. This effect was observed for supra- and subliminal primes and restricted to pictorial primes (in comparison to words). Finally, Ruch et al. could show that subliminally presented information can also impact decision-making. In a first phase, faces were presented subliminally along with written high- or low-wage occupations. These faces were presented again in a second phase, this time supraliminally and either with congruous or incongruous occupations in comparison to the first presentation. A later recall phase showed that both the previous sub- and supraliminally presented information biased participants' decision on the incomes of the depicted person.

A further relevant question in the realm of consciousness research is how stimuli get access to awareness and, in particular, how specific stimulus attributes facilitate this process. Based on previous findings indicating a hemisphere-specific processing of spatial frequencies, Piazza and Silver set out to test whether the awareness of spatial frequency information also differs between the two hemispheres. Using binocular rivalry to approach this question, the authors demonstrate that the visual system's classification of high and low spatial frequencies, and therefore the hemisphere preferentially processing the given frequency, is dependent on other, concurrently presented spatial frequencies. This indicates that a relative rather than an absolute processing of spatial frequencies contributes to hemispheric differences in perceptual selection. Another popular technique to study the access to awareness is breaking continuous flash suppression. Noel et al. applied this technique to investigate the preferential processing of self-relevant stimuli. While they did not observe such a preference for self- vs. non-self-related words, they found that participants' response criterion for the categorization of these words (i.e., self vs. non-self) was dependent on an acoustic signal administered either within or outside the peripersonal space. However, a common observation in studies using breaking continuous flash suppression is the high variability between participants. What is more, as Gayet and Stein demonstrate, the magnitude of reaction time differences between conditions is highly correlated with each individual's overall suppression times. As a remedy, the authors advocate the usage of a simple latency normalization method, which also yields reaction time distributions that are better suited for parametric testing.

Besides focusing on specific stimulus attributes that facilitate awareness, one can also ask in a broader sense how consciousness develops over time. Is the transition between unconscious and conscious states a gradual or dichotomous phenomenon? This is one of the long-standing questions that has steered heated debates in consciousness research. Using backward masking with word targets, Kiefer and Kammer varied the context by the modulation of the task and the mask type. From their results, the authors concluded that the emergence of awareness is neither purely gradual nor dichotomous but rather depends on the specific parameters of the task and the type of mask. In the context of social interactions, Kojima et al. were specifically interested in how people become aware of the presence of others. Measures of turn-taking and movement synchrony that were assessed during a social interaction paradigm indicated that the awareness of the other's presence was reciprocally co-regulated by both agents. At the neural level, the identification of brain processes that are related to or even causally determine conscious experiences has received attention under the term “the neural correlates of consciousness.” Measuring event-related potentials (ERPs) during a backward masking paradigm, Fu et al. addressed the question whether visual awareness is related to a visual awareness negativity (VAN). The authors found that ERP components were related to visual awareness for color photographs but not for line drawings. Furthermore, the VAN varied with visual awareness in a linear fashion while positive late potentials changed in a non-linear way, indicating that different ERP components are related to different types of visual awareness. In their review paper, Gallotto et al. provide a basic overview of neural oscillations and ways to measure them. They also emphasize that the distinction between the neural prerequisites, substrates, and consequences of conscious experience remains a major challenge for future research. Also with respect to the general time course of consciousness, many aspects have been left unclear to date, as pointed out by Aru and Bachmann. These open questions are particularly related to the form of the functions that describe how preconscious content accesses consciousness but also how a conscious representation can decay again. The authors particularly point toward the importance of the context, which is in line with the aforementioned findings by Kiefer and Kammer.

We received two studies addressing the question to what extent rules and regularities can be learned unconsciously. Huang et al. used ERPs to explore the impact of the response-stimulus-interval (RSI) on the transfer of knowledge of abstract implicit rules. Only at the longer of two RSIs, participants were able to acquire abstract implicit knowledge. Furthermore, the results suggest that amplitude variations of the N200 and P300 components of the ERP may be useful for detecting transfer-related effects. Esser and Haider investigated how unconscious knowledge becomes conscious knowledge in the serial reaction time task (SRTT). Regular (i.e., in line with the rule) and deviant trials (i.e., violating the rule) were either presented in mini-blocks or randomly mixed. While the degree of implicit knowledge, as assessed on the basis of a wagering task, was not affected by the presentation order, the subjectively experienced fluency was higher for the presentation in mini-blocks. More explicit knowledge was gathered for longer mini-blocks. The authors interpret their findings in light of the unexpected event hypothesis, according to which explicit knowledge arises from the observation of one's own changes in behavior that, in turn, are based on implicit learning.

In their pupillometry study, Chen at al. asked how the conscious representation of visual input can be dissociated from its consequences. They presented looming spheres on the screen such that they would either collide with the observer or miss the observer's head by a small margin. Participants had the task to either judge the size of the stimulus or to decide whether the stimulus would have collided with them. In all experiments (with the exception of the first one), participants could not distinguish between collisions and near-misses. The results showed that participants judged the size of the colliding stimuli as being bigger than the near-miss stimuli, and pupil constrictions turned out to be greater for the colliding stimuli. The authors conclude that threatening stimuli may influence visual perception without necessarily evoking a conscious representation of the threat.

In addition, one general commentary and one original research article were concerned with the scope of unconscious high-level cognitive functions. In their commentary, Goldstein and Hassin follow up on the debate about the “Yes It Can” (YIC) principle (Hassin, 2013; Hesselmann and Moors, 2015). According to YIC, unconscious processes can carry out every fundamental high-level function that conscious processes can perform. As the authors point out, one of the implications of YIC is that the search for a “holy grail” —i.e., the function that only consciousness can do—is the wrong way to go. Instead, understanding what it means to be human would be better achieved by understanding how unconscious processes pursue functions that only they can pursue. In their study, Garrison and Handley tested the hypothesis that “unconscious thought” (Dijksterhuis and Nordgren, 2006) is distinct from intuitive processes, and thus might be rational. The authors manipulated participants' reliance on the experiential versus rational system (Epstein, 1994) and found that a period of distraction facilitated outcomes independently from these two processing modes. They also manipulated unconscious thought (during a distraction phase) toward solving a logical reasoning problem, and observed that unconscious thought was superior at this analytical task, suggesting that unconscious thought may be rational. As the authors point out, however, the concept of “unconscious thought” itself remains controversial (Nieuwenstein et al., 2015).

The main goal of this Research Topic “Transitions between consciousness and unconsciousness” was to provide a snapshot of the current state of affairs in this research area. The final collection of 18 articles does exactly that and provides an overview of current trends and opinions, as well as perspectives on theoretical and methodological questions. As pointed out by two of us in a perspective article, research on conscious and unconscious processes is characterized by a large diversity of methods, measures, statistical analyses, and concepts. The same holds true for this collection. We hope that the reader will find the collected articles both informative and thought-provoking, and that this Research Topic will stimulate the scientific debate.

## Author contributions

All authors listed have made a substantial, direct and intellectual contribution to the work, and approved it for publication.

### Conflict of interest statement

The authors declare that the research was conducted in the absence of any commercial or financial relationships that could be construed as a potential conflict of interest.
